# Time to Change Theory; Medical Leech from a Molecular Medicine Perspective Leech Salivary Proteins Playing a Potential Role in Medicine

**DOI:** 10.34172/apb.2021.038

**Published:** 2020-07-26

**Authors:** Amir Shakouri, Uwe Wollina

**Affiliations:** ^1^Drug Applied Research Center, Tabriz University of Medical Sciences, Tabriz, Iran.; ^2^Department of Dermatology and Allergology, Academic Teaching Hospital Dresden-Friedrichstadt, Dresden, Germany.

**Keywords:** Leech saliva, Medical leeches, Protein, Anti- cancer, Cancer

## Abstract

Followed by developing modern medicine, leeches did not have extensive use as before; however, in the late 19th century, they were still used in most countries all over the world. Thus far, leeches were utilized in treating various diseases like skin disorders, arthritis, and cancer. In Egypt, using leeches for treatment dates back to early 1500 BC. A medical leech’s salivary glands involve over 100 bioactive proteins and the salivary gland secretion contains bacteriostatic, analgesic, and anticoagulation influences; with resolving activity, it causes microcirculation disorders elimination, restoring the hurt vascular permeability of organs and tissues, removing hypoxia, decreasing blood pressure and detoxifying the organism by antioxidant paths. The current work reviews the innovative treatment with medical leech, especially proteins in leech saliva extraction (LSE) with high potential in medicine. The virtue of salivary gland secretions which are proteinaceous enzymes, leech acts on various diseases such as venous congestion in reconstructive and plastic surgery, osteoarthritis, cardiovascular diseases caused by blood coagulation disorders, pain management, priapism, macroglossia, cancer complications, wounds and many other. To confirm the potential therapeutic impacts of leech treatment, more studies are required in more extensive areas with more exact methodologies.

## Introduction

Medicinal leech therapy (MLT) or hirudotherapy is an integrative and complementary treating technique utilized with blood-sucking leeches. A leech or more are connected to the problematic region’s skin to obtain potential efficacies of the leech’s saliva secreted when the leeches are nourishing. MLT was utilized over the centuries and the word leech was rooted in “laece” (physician). The initially recorded usages were related to ancient Egypt. Furthermore, there are numerous references to MLT in Arabic, Chinese, Anglo-Saxon, Roman, and Ancient Greek medical records. In Europe in the 17th century, MLT had the most extensive usage area.^1,2^ In the 1900s, less attention was taken by medical professionals; however, in the last 30 years, MLT has become a key component in most scientific research.^3,4^ Leeches live in freshwater and are hermaphrodite, segmented, and carnivorous worm’s sensitive to ambiances in the water, light, touch, sound, heat, and different chemicals. They are multi-segmented, such as “brain parts”, each part with various organs like testicles and ganglions. Creeping and adherence are carried out by two sucker parts; three jaws are included in the anterior with numerous teeth. They normally bite the host’s warm parts to suck the blood with regular contractions. Nourishing typically takes about 40 minutes and 10–15 mL of blood is digested by a leech per feeding.^5,6^ Digestion is performed by numerous enzymes as well as mutual microorganisms like *Pseudomonas hirudinia* and *Aeromonas hydrophila*. Previously, MLT was examined and is extensively utilized after reconstructive, plastic, and microsurgical applications, in cardiovascular diseases, postphlebitic syndrome, deep vein thrombosis, tinnitus, complications of diabetes mellitus, chronic and acute otitis, and in relieving the osteoarthritis pain.^7^ Over 600 leech species exist; however, *Hirudo medicinalis*, *Hirudo nipponia, Hirudoquin questriata*, *Hirudotroctina*, *Poecilobdella granulosa*, *Hirudinaria manillensis*, *Hirudinaria javanica*, *Macrobdella decora*, and *Haementeria officinalis* are the most commonly used in the world.^8^

In numerous studies, it was found that different bioactive molecules are involved in the leeches’ secretions. Over 100 molecules and their action modes were recognized; however, further awaiting explorations exist. These molecules possess anti-inflammatory, analgesic, platelet inhibitory, anticoagulant, anti-cancer anti-metastasis, and thrombin regulatory functions, along with extracellular matrix antimicrobial and degradative influences. It is stated that further investigations are required for more indications probably emerging by currently elucidated effect mechanisms. This paper aimed to collect information regarding medical leech saliva extraction (LSE), to offer a general vision, and to consider the action modes extensively.

### Medical leech saliva proteins

The chief therapeutic advantages are not resultant from the blood taken over the biting (while this probably offers dramatic relief at first); however, they are derived from the vasodilator and anticoagulant included in the leech saliva. Over 100 bioactive proteins are contained in a medical leech’s salivary glands with antiedematous, analgesic and bacteriostatic effects^9^; it has resolving activity, causes to microcirculation disorders elimination, restoring the hurt vascular permeability of organs and tissues, hypoxia (oxygen starvation) elimination, blood pressure reduction, increased immune system activity, detoxified organism through antioxidant path, relieving it from the hostile complications like strokes and infarct, and the organism’s bioenergetic status improvement.^10^ A summary of proteins that exist in leech saliva placed in Table 1.

**Table 1:** Leech saliva contain several main proteins which have multi-functional activity in medicine

**Main proteins of LSE**	**Molecular weight (Da)**	**Protein Reaction**
Hirudin	6970	Hirudin is a potent thrombin-specific protease inhibitor. Inhibits blood coagulation by binding to thrombin
Hyaluronidase	55090	hydrolase activity, acting on glycosylic bonds
Eglin C	8100	Anti-inflammatory. Inhibit the activity of chymotrypsin chymase, subtilisin, elastase and cathepsin G
Hirustasin	5878	Acts as an inhibitor of tissue kallikrein, trypsin, chymotrypsin and neutrophil cathepsin G
Factor Xa inhibitor	15225	Inhibits the activity of coagulation factor Xa by forming equimolar complexes

### Hirudin 

**Figure 1:**
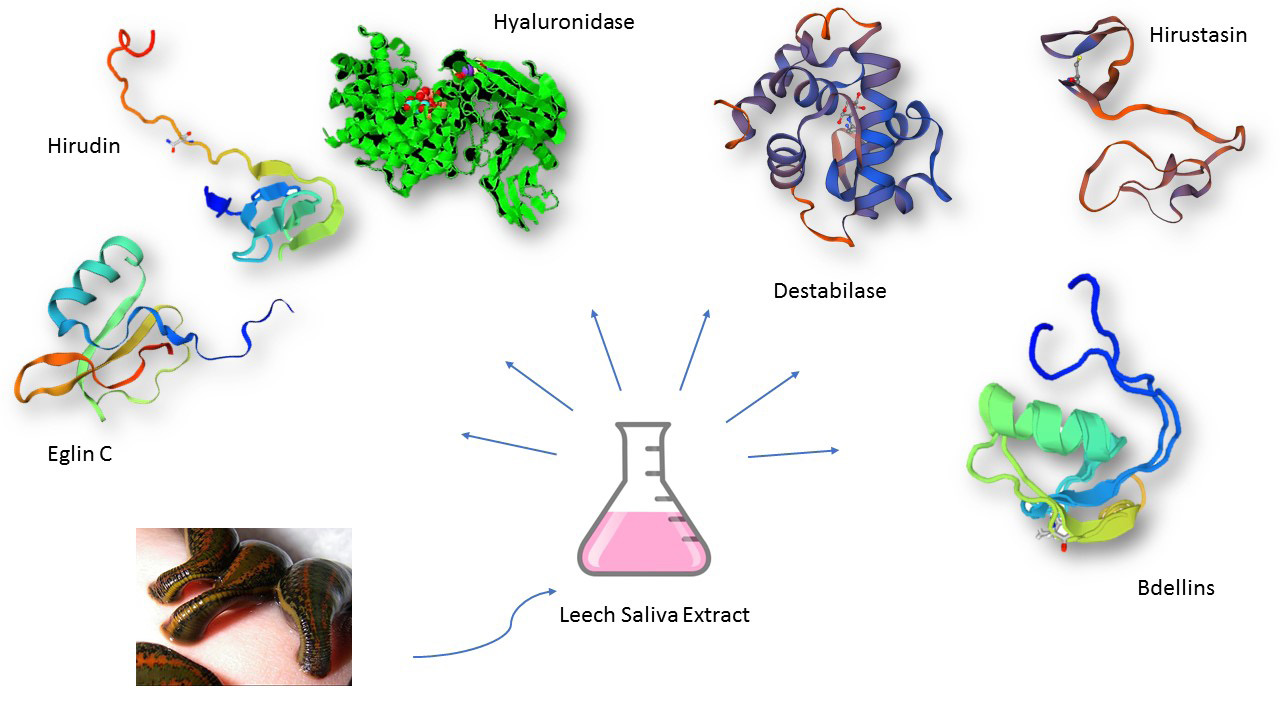
Medical leech saliva contains wide range of proteins and enzymes which have multifunctional effect in diseases.

Hirudin is an active component in the leeches’ salivary gland secretion and acts as an effective anticoagulant (blood thinner). It is a 7.1-kDa protein irreversibly binding to thrombin resulting in the use of antithrombin activity and active thrombin. A robust consensus exists regarding hirudin as a therapeutic alternative to heparin as a result of fewer adverse impacts and its greater anticoagulant activity. By binding to thrombin, it hinders blood coagulation. In the equimolar thrombin-hirudin complex, all the thrombin’s biological functions are blocked. Therefore, not only hirudin avoids fibrinogen clotting but also hinders other thrombin-catalyzed hemostatic reactions like the clotting factors V, VIII, and XIII activation and the activation of thrombin-induced platelet. Hence, through the instantaneous inhibition of thrombin created after activating the coagulation system, the positive response on prothrombin activating typically resulting in accelerated creation of thrombin, is hindered while delaying the thrombin creation. Therefore, coagulation is retarded or completely prevented relying on the hirudin concentration in blood.^11,12^ In Figure 1 previous research of LSE demonstrated that it contains a wide range of active proteins with diverse molecular weight.

### Hyaluronidase

It enables the diffusion and penetration of pharmacologically active materials into the tissues, particularly in joint pain and possesses antibiotic features. Hyaluronidase (Hyal) can be used to realize various complications associated with hyaluronic acid (HA).^13^ It involves some catalyst groups of that cleave HA. This enzyme is found in various human tissues and in animal pathogenic organisms, venoms, and cancers. The CD44 receptor present in a cell membrane is often increased by destructive cancer cells. This receptor plays the role of an exact receptor for HA, and it is recognized that HA motivates spreading, migration, attack, and cancer cells’ metastasis.^14^

### Destabilase

It is an enzyme possessing glycosidase activity showing both fibrinolytic and antibacterial actions. This enzyme contains different isoforms with various capabilities, and different leech species extracts it. Destabilase possesses a main degradative action on stabilized fibrin. Moreover, it also needs to be assessed as an anticoagulant agent.^11^

### Hirustasin

Hirustasin is a 55 amino acids peptide, purified from medical leech saliva. Hirudo antistasin is serine protease inhibitors which is called Hirustasin and fundamentally it is able to bind with tissue kallikrein and also it precisely hinders the blood coagulation factor Xa. Hirustasin seems to be one of the most elementary proteinase inhibitors existing in leech saliva. Hirustasin has other activities such as inhibitor of trypsin, chymotrypsin and neutrophil cathepsin G.^15^ Hirustasin, able to prevent tissue kallikrein which is valuable as a novel inhibitor of tissue kallikrein and playing important role in diseases. The tissue kallikrein/kinin system contributes to the maintenance of normal blood pressure. Tissue kallikrein has also been exist in a colorectal cancer cell line and in breast cancer cells. As a strong inhibitor of tissue kallikrein, hirustasin could be applied as an antimetastatic protein with no anticoagulant effects.^16^

### Bdellins

Protease inhibitors logically exist in living organisms, as well as animals’, plants, and bacteria. They have wide range of activities in many physiological procedures and play a significant role in biological activity of animal’s venom. To inhibit clotting throughout blood feeding from a host, animals have established several mechanisms to prevent blood coagulation. Among the inhibitors involved in coagulation, protease inhibitors like bdellin are the most essential anticoagulants currently described from medical leeches which is called bdellins with anti-inflammatory effect, it hinders plasmin, trypsin, and acrocin. The bdellins display strong inhibitory activity towards the trypsin like proteinase acrosin present in the acrosomes of spermatozoa.^17^

### Eglin C

Eglin c is a natural protein from the leech *Hirudo medicinalis* which contains 70 amino acids. It powerfully prevents the chymase, alpha chymotrypsin, subtilisin, elastase, and cathepsin G activity.^18,19^ It has thus attracted particular attention as a possible therapeutic agent against various pathogenic elastic tissue agents, blood clotting disorders and inflammatory processes. Furthermore, Eglin might be a valuable for therapeutic approaches in diseases induced by neutral granulocytic proteinases.^20^

### Factor Xa inhibitor

It hinders the coagulation factor Xa activity. The coagulation cascade is broken by factor Xa inhibitor with a straight anticoagulant effect. It plays a vital role in medical leech therapy of rheumatoid arthritis and osteoarthritis. Furthermore, as stated before, factor Xa is directly inhibited by antistasin, and possible anticoagulant effects are included in ghilantens, LDTI, C1 inhibitor, and Eglin, possibly through direct and/or indirect hindering of coagulation factors.^21^

## Medical use of leech therapy in clinical use 

Leech therapy is mostly utilized in the localized venous congestion settings related to ﬂap surgical replantation and reconstructions.

## Arthritis

The saliva of leech contributes to treating arthritis. Some materials and compounds exist in its saliva contributing to reducing inﬂammation in a joint. Among which are Eglin C and Bdellins acting as anti-inﬂammatory materials. In addition to anti-inﬂammatory components, its saliva also contains an anesthetic component alleviating the pain felt in the joint. It also includes a histamine-like material acting as a vasodilator. Another component of the saliva of the leeches is acetylcholine, which is also a vasodilator.^22,23^

## Venous congestion

It was proven that leech therapy helps patients with venous diseases. It can contribute to reducing the swelling and the pain, as a result of varicose veins, and assists dissolve blood clots. Though, leech therapy is not operative in illnesses triggered by deficient vessel dilation and inadequate valves.^24^

## Vascular diseases 

Recently, leech therapy is used for curing vascular disorders. The leech saliva contains more than 100 very beneﬁcial bioactive materials. Among these components is hirudin acting as an anticoagulation agent. Another component is Calin also inhibiting blood coagulation. The destabilase is a component dissolving ﬁbrin clots and inhibiting thrombus formation.^25^ The saliva of leech also has a factor Xa inhibitor restraining the coagulation Factor’s coagulating effect. It also contains hyaluronidase improving the interstitial ﬂuid’s viscosity. Regarding a vasodilating effect, it contains histamine-like, acetylcholine materials and carboxypeptidase-A inhibitors.^26^ Leech saliva only contains some very useful components working in the background to reduce the blood viscosity for promoting better ﬂow. It was prone that blood with a thick consistency causes clot creation and increments the blood pressure of a person. These clots are able to travel to various parts of the body and can block a vessel, thus resulting in a heart attack or stroke. Thick blood leads to a risk exterminating the distal, particularly enough oxygenated blood and the required nutrients will not be received to the tips of the toes and ﬁngers. Hence, the anticoagulation component in the saliva of a leech is important and with all these components acting together, a considerable enhancement will be obtained in the patient’s vascular status.^27^

## Anti-microbial activity 

It was shown that only two key molecules, chloromycetin, and destabilase possess antimicrobial activity, so far. As previously mentioned, destabilase possesses β-glycosidase activity straightly disrupting β1–4 bonds imperative in the peptidoglycan layer of the bacterial cell walls. It is obvious that this action is the same as lysozyme regularly existing in lachrymal fluid and human saliva. Other investigations indicated that not only antimicrobial activity relies on glycosidase enzymatic activity, but also it relies on non-enzymatic components.^28^ Even a dose-dependent bacteriostatic effect is shown by the desaturated form destabilase on Staphylococcus aureus, Escherichia coli, and Pseudomonas aeruginosa. Chloromycetin is a potent antibiotic existing in leech secretions; however, there are limited data regarding this molecule. Furthermore, theromyzin, peptide B and theromacin, were isolated as antimicrobial peptides.^29^

## Pain management

Leeches are utilized in different origins’ pain syndromes. The pain relief is fast and sometimes takes a long time. Reports demonstrated that leech therapy could be beneficial in severe cancer pain. There is argument in studies on osteoarthritis for symptomatic enhancement through leech therapy by anti-inflammatory and analgesic effects.^30^

## Leeches in cancer

**Figure 2:**
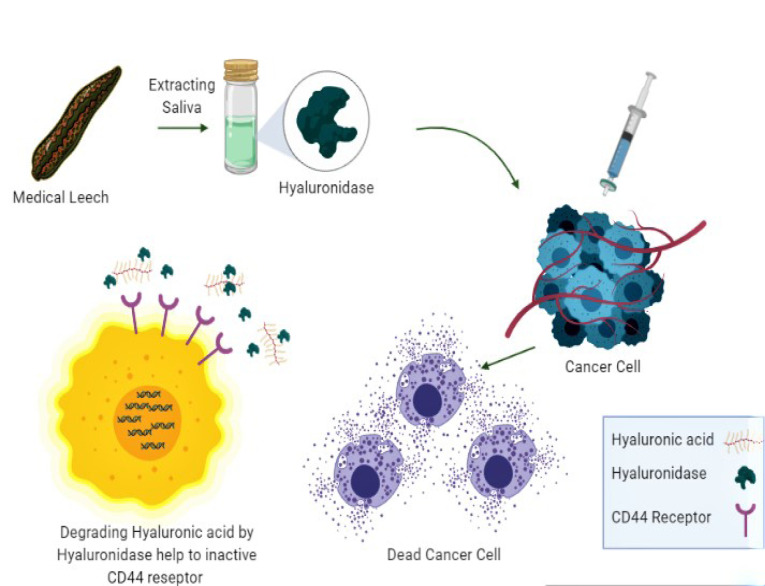
Activity of Hyaluronidase in Cancer. Degrading hyaluronic acid by hyaluronidase, inhibiting CD44 activation and after a while mRNA of VEGF reduced and it helps to reducing angiogenesis of cancer cells.

After surgery, a patient with basal cell carcinoma, underwent leech therapy for 9 months and good results were found based on accomplishing blood circulation across the flap. The medical leeches are effective in relieving venous congestion of a free forearm flap followed by reconstructing in a patient with intraoral carcinoma. The leech’s salivary gland secretions involve antimetastatic activity. A protein termed antistasin is included in the leech’s saliva which hinders lung cancer colonization. There are the anti-proteolytic, platelet aggregation inhibitors, and anticoagulants enzymes in the secretions. Furthermore, anti-tumor activity is included in other components like hyaluronidase. It is conceived that by degrading the hyaluronic acid-CD44 contact the hyaluronidase anticancer activity may happen to some extent through pro-tumorigenic immune cell inhibition into the tumor stroma.^31^ Figure 2 shows LSE application in cancer.

## Conclusion

Medical leech therapy possesses a long history; however, its effect mechanisms are currently being clarified. By a leech biting, collagenase and hyaluronidase can access to the blood vessels and tissues; vasodilatation happens by acting the histamine-like molecules; then, kinin activity, platelet functions, and the coagulation cascade are prevented while inflammatory reactions are repressed. Furthermore, antimicrobial and analgesic effects are also found. Joint illnesses such as epicondylitis and osteoarthritis, flap surgery (skin grafting), and extremity vein diseases are the main indications for MLT. For periorbital hematoma and soft-tissue, purpura fulminans, macroglossia, prosthetic syndrome, ecchymosis and penile replantation, MLT is also effective. Moreover, anticoagulants attained from leeches are utilized for infectious myocarditis and peripheral arterial occlusion. MLT is not suggested in cases with hemorrhagic diathesis, leukemia, bone marrow suppression, anticoagulant therapy, cirrhosis, dialysis, radiotherapy, and chemotherapy.^32^

It can be concluded that MLT is an effective traditional method with robust biochemical actions. Though bioactive materials and action modes are still under more exploration, they have obvious utility in definite medical circumstances. Potential and indications complications should be assessed such as application frequency and antibiotic prophylaxis, and dosage and delivery time depending on the patient and opinion of physician. It should be stated that MLT is not a therapy method alone; however, it can be a vital section of a multidisciplinary method.^21^ Regarding Leech therapy, efforts need to be made in extracting proteins from saliva and formulate them in nanocarrier in terms of novel development in cancer study.

## Future prospects of leech therapy

Leech therapy with a long history goes from well-accepted and popular technique to dropping out of favor. In comparison with other natural therapy and complementary methods, Leech Therapy can be trained fairly fast to decrease the complications caused by the unnecessary use of synthetic drugs. Today, research is carried out in different areas to define the leeches’ therapeutic role in different disease circumstances such as female and male sterility, diabetes, lupus erythematosus, prostate diseases, and many others.

## Ethical Issues

This article does not contain any studies with human participants or animals performed by any of the authors.

## Conflict of Interest

The authors declare that they have no conflict of interest.

## Acknowledgments

This information is a result of the MSc thesis registered at Tabriz Medical Science University. This study was conducted at Immunology Lab of Drug Applied Research Center of Tabriz medical science University. The authors thank all staff of the Immunology Lab.
